# Effects of the COVID-19 pandemic on cardiovascular disease financing in Indonesia (JKN claims data analysis 2019–2020)

**DOI:** 10.3389/fpubh.2023.1148394

**Published:** 2023-03-31

**Authors:** Wahyu Pudji Nugraheni, Ekowati Retnaningsih, Rofingatul Mubasyiroh, Tety Rachmawati

**Affiliations:** ^1^Research Center for Public Health and Nutrition, Research Organization for Health, National Research and Innovation Agency, Bogor, Indonesia; ^2^Regional Research and Development Agency South Sumatra Province, Palembang, Indonesia

**Keywords:** COVID-19, cardiovascular disease, financing, National Health Insurance, Indonesia

## Abstract

The disease burden related to non-communicable diseases is a major public health problem in Indonesia. About one-third of all deaths in Indonesia are caused by cardiovascular disease. This study describes the cost of cardiovascular disease from claims data for Advanced Referral Health Facilities at BPJS Kesehatan before and during the COVID-19 pandemic. We analyzed claims data on the National Health Insurance system managed by BPJS. The data comes from referral health facilities throughout Indonesia in 2019 and 2020. Cardiovascular service claims data by sex and age group were analyzed descriptively and with different tests between years. There was a decrease in the number of patients accessing cardiovascular services at referral health facilities for all genders, age groups, and types of main diagnoses, by around 27.8%, from 933,017 (2019) to 673,801 (2020). There was a significant decrease in total claims for all types of cardiovascular disease during the COVID-19 pandemic compared to before, especially the reduction in aneurysms and aortic dissection (40.2%) and hypertensive heart disease (39.6%). The decline also occurred in all sexes and age groups, with an average percentage decline of 31.2%. Our findings show that the reduction in the cost of cardiovascular disease claims goes hand in hand with the decrease in the number of cardiovascular patient visits. To guarantee treatment for cardiovascular disease patients during the COVID-19 pandemic, BPJS can maximize the telemedicine services that have been built. The organizer of the National Health Insurance program in Indonesia has developed a JKN mobile application that has the potential for telemedicine services guaranteed by JKN. On the other hand, BPJS needs to limit promotive and preventive budgets related to CVD so that it does not become a potential catastrophic financing.

## Introduction

Globally, non-communicable diseases (NCDs) contribute to more than 60% of disability-adjusted life years (DALYs), 70% of deaths, and more than 80% of years of living with a disability. Cardiovascular diseases, which comprise ischemic heart disease, stroke, heart failure, peripheral arterial disease, and other heart and vascular conditions, represent 24% of NCD-associated DALYs and are the leading cause of global death and a major contributor to decreased quality of life (1, 2). In Indonesia, the burden of the disease related to non-communicable diseases is a major public health problem. About one-third of all deaths in Indonesia are caused by cardiovascular disease, with stroke and coronary heart disease being the main causes (3). In the last 10 years (2007–2017), there has been an increase in the burden of non-communicable diseases from 63% to 72%.

COVID-19 is a pandemic disease characterized by a respiratory infection caused by the coronavirus. It has spread worldwide since December 2019. The development of COVID-19 cases in Indonesia has been relatively fast since the first positive case was detected on 2 March 2020 (4). Patients with cardiovascular disease are more susceptible to infection and exacerbation of SARS-CoV-2 (5). Hypertension, diabetes, and atherosclerotic cardiovascular are independently associated with severe COVID-19 and, together with age and male sex, can be informative for predicting the risk of severe COVID-19 (6). In most cases, cardiovascular disease is a preventable condition that may be managed by reducing or eliminating a number of risk factors, such as hypertension, diabetes, dyslipidemia, obesity, smoking, insufficient physical activity, a poor diet, and alcohol use. In developing countries, lifestyles have changed dramatically due to rapid urbanization, which is characterized by an increase in poor diets and the adoption of sedentary lifestyles (7, 8).

The pandemic has harmed health services, including the difficulty of access to health facilities, lack of health personnel, and the social restrictions imposed. The movement of people has also become limited, not only healthy people but also sick people. One of the services that have fallen drastically is diagnosing and treating non-communicable diseases, with 69% of countries reporting this disorder (9). It is evident from an online survey in Indonesia in August–October 2020 that 20.4% of respondents reduced their routine use of health services (10).

The Social Security Administering Body, which was established through Law no. 24 of 2011, is a form of National Health Insurance providing Universal Health Coverage for all Indonesian people (11). The National Health Insurance Program is for all Indonesian residents, including foreigners who have worked for a minimum of 6 (six) months in Indonesia, and who have paid contributions. The number of participants reached 224,149,019 people in 2019 and 222,461,906 people in 2020 (12). This agency manages both outpatient and inpatient health services at first-level facilities and advanced referral facilities registered with Social Security Administering Body For Health (BPJS Kesehatan). The first-level facilities include Puskesmas, doctor's practice, Pratama clinic, and class D Pratama hospital. Advanced Level Referral Health Facilities include main clinics, general hospitals, and special hospitals. Payment for health services at hospitals uses the Indonesian Case Base Groups (INACBGs) system based on submitting claims from hospitals, both outpatient and inpatient (13).

Cancer and heart disease occupy the first and second catastrophic costs in National Health Insurance Program financing in Indonesia. In 2019, around 18.5% of the total National Health Insurance Program financing was catastrophic health financing (14, 15). During the pre-COVID-19 period, the health social security fund administered by the Social Security Administering Body experienced a budget deficit, and in the end, the government had to issue an allocation to cover the deficit (16). One of the contributors to the deficit is the adverse selection phenomenon, especially in people with catastrophic diseases, such as cardiovascular diseases. People with catastrophic diseases tend to register and take advantage of services managed by BPJS. Since it is well known that catastrophic diseases require large costs and long-term treatment (17). With no difference in financing pattern before the COVID-19 period (18), the Social Security Administering Body experienced a financial surplus during the pandemic (19).

The overall impact of the National Health Insurance Program contribution adjustment and low healthcare utilization has resulted in a financial surplus of the National Health Insurance fund. The National Health Insurance administrator management still needs to anticipate the period when the government announced that the COVID-19 pandemic has ended since that is the start of the COVID-19 financing under the National Health Insurance scheme (20). Considering the characteristics of cardiovascular diseases as catastrophic diseases and pandemic conditions, we would like to examine or know the pattern of cardiovascular disease financing in the period before and during the COVID-19 pandemic.

## Materials and methods

### Study design

This cross-sectional study was a secondary data analysis using the National Health Insurance claims data to describe patterns of visits and cardiovascular claims under the National Health Insurance Scheme.

### Setting and data source

We analyzed data claims of the National Health Insurance managed by the Health Social Security Administration. We collected the data on outpatient and inpatient claims (in IDR) from all Advanced Level Referral Health Facilities (FKRTL) that collaborated with BPJS in Indonesia (34 provinces) in 2019 (before COVID-19) and 2020 (during COVID-19). In 2019, there were a total of 2,459 FKRTL and a total of 2,507 FKRTL that collaborated with The Social Security Administering Body (21). These claims data were payments by The Social Security Administering Body to service provider hospitals based on the Indonesia Case-Based Group (INA-CBG), which was equivalent to the local Diagnosis-Related Group (DRG). Expensive drugs, such as anticancer drugs, were not bundled with the goal of better predicting their use. Patients received full coverage for drugs identified in the national formulary at public hospitals (22).

### Participants

The participants in this study were all patients suffering from cardiovascular diseases and utilizing BPJS services in 2019 and 2020. These patients were scattered among all hospitals that cooperate with BPJS in Indonesia. Patients consisted of all genders (male and female) and all age groups (0–80+ years).

### Variable

Our data are not in the form of individual data but rather aggregate data for groups of individuals according to sex, age group, and type of disease. The claims data that we analyze are service claims data (main diagnosis) of cardiovascular disease based on the International Statistical Classification of Diseases and Related Health Problem Tenth Revisions (ICD-X) code, which includes aortic aneurysm and dissection (I71), atrial fibrillation (I48), hypertensive heart diseases (I11), hemorrhagic stroke (I619), ischemic stroke (I64), and ischemic heart diseases (I20, I21, I22, I23, I24, and I25). These disease codes are included in the criteria for diseases of the circulatory system (https://icd.who.int/browse10/2019/en#/).

### Data management and analysis

Cardiovascular disease claims data were analyzed descriptively by adding up patients who had access to FKRTL according to sex characteristics, age group, and type of disease in 2019 and 2020 (Table 1). We calculated the difference in claims used by patients according to the characteristics of the type of disease in 2019 and 2020. From this difference in claims, we calculated the percentage and tested the difference in claims using the Wilcoxon test with a value limit of α = 0.05 (Table 2). Data claims were not normally distributed, so to enforce the different claim tests based on characteristics, we use Wilcoxon analysis (23). We calculated the difference in claims used by patients according to sex and disease characteristics in 2019 and 2020. From the difference in claims, we calculated the percentage (Table 3). This percentage was calculated as the difference in claims (in rupiahs) divided by the total claims in 2019 (in rupiahs), multiplied by 100%. All analyses were performed using the SPSS software.

**Table 1:** Characteristic of cardiovascular disease patients in National Health Insurance claims, Indonesia, 2019–2020.

**Characteristics**	**Number of patients**		**Reduction**	
	**2019**	**2020**	**Number**	**%**
**Sex**				
Male	494,412	363,585	130,827	26.5
Female	438,605	310,216	128,389	29.3
**Age group**				
0–4	1,048	620	428	40.8
5–9	539	265	274	50.8
10–14	730	445	285	39.0
15–19	2,121	1,380	741	34.9
20–24	3,334	2,637	697	20.9
25–29	5,840	4,785	1,055	18.1
30–34	11,376	8,850	2,526	22.2
35–39	24,359	18,342	6,017	24.7
40–44	48,150	36,543	11,607	24.1
45–49	85,835	62,978	22,857	26.6
50–54	129,857	96,609	33,248	25.6
55–59	155,010	113,293	41,717	26.9
60–64	156,836	114,463	42,373	27.0
65–69	129,558	92,721	36,837	28.4
70–74	84,251	58,626	25,625	30.4
75–79	57,554	36,989	20,565	35.7
80+	36,619	24,255	12,364	33.8
**Type of diseases**				
Ischemic heart diseases	387,919	289,021	98,898	25.5
Ischemic stroke	206,443	152,906	53,537	25.9
Haemorrhagic stroke	56,374	45,568	10,806	19.2
Hypertensive heart diseases	246,104	158,509	87,595	35.6
Atrial fibrillation	34,709	26,769	7,940	22.9
Aortic aneurysm and dissection	1,468	1,028	440	30.0
**Total**	**933,017**	**673,801**	**259,216**	**27.8**

**Table 2:** Differences in total cardiovascular disease claims in Indonesia, 2019–2020.

**Primary diagnose**	**Total of claims**		**Claims difference**		
	**2019 (billion)**	**2020 (billion)**	**IDR (billion)**	**%**	***p*** **-value**
Aortic aneurysm and dissection	37.367	22.327	15.040	40.2	<0.001
Atrial fibrillation	171.414	125.794	45.620	26.6	<0.001
Haemoraghic stroke	496.192	385.434	110.758	22.3	<0.001
Hypertensive heart diseases	373.755	225.717	148.038	39.6	<0.001
Ischemic heart diseases	3,378.107	2,311.414	1,066.694	31.6	<0.001
Ischemic stroke	1,338.802	978.983	359.819	26.9	<0.001
Average	965.940	674.669	290.995	31.2	

**Table 3:** Total cardiovascular disease claims by gender and type of disease in Indonesia, 2019–2020.

	**Sex**	**Number of patients**		**Total claims (billion IDR)**		**Difference claims**	
		**2019**	**2020**	**2019**	**2020**	**IDR (billion)**	**%**
Ischemic heart diseases	Male	231,313	175,084	2,435.926	1,663.207	772.719	31.7
	Female	156,606	113,937	942.182	648.207	293.974	31.2
Ischemic stroke	Male	111,046	82,970	729.670	536.071	193.599	26.5
	Female	95,397	69,936	609.132	442.912	166.220	27.3
Haemoraghic stroke	Male	29,211	23,566	278.990	212.799	66.191	23.7
	Female	27,163	22,002	217.202	172.635	44.567	20.5
Hypertensive heart diseases	Male	106,282	69,133	171.170	102.552	68.618	40.1
	Female	139,822	89,376	202.585	123.165	79.420	39.2
Atrial fibrillation	Male	15,531	12,153	78.460	57.456	21.004	26.8
	Female	19,178	14,616	92.954	68.338	24.616	26.5
Aortic aneurysm and dissection	Male	1,029	679	29.928	16.401	13.526	45.2
	Female	439	349	7.440	5.926	1.513	20.3

## Results

The data analyzed in this study are claims data for several types of cardiovascular disease in 2019 and 2020 from all FKRTLs in Indonesia. Table 1 shows the number of patients who accessed cardiovascular services at FKRTL; in 2019, it was 933,017 people and decreased to 673,801 patients in 2020. In 2019 and 2020, more male patients accessed cardiovascular services in hospitals than female patients. When viewed from the percentage decrease, women (29.3%) are slightly higher than men (26.5%).

In 2019, the number of patients aged 0–4 years was more than those aged 5–14 years. In these 2 years, the number of patients will increase with increasing age upto 64 years. The number of patients will decrease again at the age of 65 years to more than 80 years. The number of patients decreased in all age groups from 2019 to 2020. However, looking at the percentage decrease in the number of patients, the 0–19 years and 70+ years experienced a 30–50% decrease.

The highest number of patients in 2019 and 2020 were cases of ischemic heart disease, followed by hypertensive heart diseases and ischemic stroke. The number of patients per type of disease decreased from 2019 to 2020. However, if we look at the percentage decrease in the number of patients, hypertensive heart diseases (35.6%) and aortic aneurysm and dissection (30.0%) patients experienced the most declines. Meanwhile, the number of hemorrhagic stroke patients decreased the least, less than one-fifth from 2019.

Ischemic heart disease and ischemic stroke dominated the largest total claims for treatment costs in 2019 (Table 2). The same pattern was also seen during the 2020 COVID-19 pandemic. There was a significant decrease in total claims for all types of cardiovascular disease during the COVID-19 pandemic compared to before the 2019 pandemic. The average decline for each of the six cardiovascular disease claims was 290,995 billion, with an average percent decrease of 31.2%. However, looking at the percentage of the largest decrease, it is in the case of aortic aneurysm and dissection, which is 40.2%, followed by hypertensive heart diseases with a 39.6% reduction in claim costs. Meanwhile, the lowest percentage decrease in total claims was in cases of hemorrhagic stroke, which did not decrease by a quarter of the cost before COVID-19 (22.3%).

Table 3 shows the difference in the cost of NCD claims by gender from 2019 to 2020. Based on the nominal cost, the biggest decrease is the treatment of ischemic heart disease in male and female patients, followed by an ischemic stroke in male and female patients. However, in the case of hypertensive heart disease, the cost reduction was more for female patients than male patients. The smallest nominal decrease was in aortic aneurysm and dissection cases in women, followed by men.

However, when viewed from the percentage of costs, the largest percentage decrease was in the cost of aortic aneurysm and dissection in men (45.2%), followed by the percentage decrease in the cost of hypertensive heart disease in men (40.1%) and in women (39.2%). The lowest percentage of reduction in financing was aortic aneurysm and dissection in women (20.3%) and hemorrhagic stroke in women (20.5%).

## Discussion

This study indicates that visits and claims of cardiovascular disease patients between before and after the COVID-19 pandemic showed a decrease, with a 31.2% decrease in claims. This decline in inpatient visits to health facilities also occurred in other countries. In Singapore, the COVID-19 pandemic was associated with a 9.3% reduction in doctor visits and inpatient visits and a 2.7% decrease in the likelihood of being diagnosed with a chronic condition (24). In Colombia, compared to 2019–2020, total emergency health services decreased from 83,925 to 71,611, with 133 per 100,000 person-months (25).

The decline in cardiovascular patient visits in Indonesia can be caused by various things, including decreased community mobility due to social distancing policies with changing policy methods ranging from Large-Scale Social Restrictions (PSBB) to Community Activity Restrictions (PPKM) with different levels in each region (26). This condition is in line with the BPJS report, which stated that there was a surplus in non-COVID-19 disease financing during the pandemic due to decreased health service utilization (20).

During the COVID-19 pandemic, National Health Insurance was in a surplus condition. This condition was not only caused by the decrease in utilization rates during the COVID-19 pandemic but also by several factors that contributed to the National Health Insurance surplus, namely adjustments to National Health Insurance rates, improvements in strategic purchasing in health services, and improvement in service quality (27, 28).

Furthermore, some people choose to be vigilant so as not to contract COVID-19 and choose to do activities and work at home, although some are less vigilant (3). This condition is also supported by the Indonesian association of specialist doctors who warn the public to postpone hospital visits if they are not in an urgent condition (29).

The COVID-19 pandemic has not only impacted public health but has also affected the Indonesian economy, education, and social life. One of the economic impacts occurred due to the implementation of PPKM and PSBB policies, namely government policies that prohibit people from congregating and carrying out activities outside the home. People are required to remain in their houses. With this regulation, community activities, employees, workers, and factory workers are forced to be sent home or reduced time for work and to terminate employment, causing unemployment (30, 31). When unemployment occurs, income automatically decreases dramatically. One of the reasons for this decline in income is the ability to pay health insurance premiums. The Health Social Security Administration data show that during the COVID-19 pandemic, the participation coverage rate dropped significantly (28).

Based on gender, the decrease in cardiovascular patient visits was more in female cardiovascular patients. More male patients access cardiovascular services in hospitals than female patients (32). Female heart disease patients are more likely to receive less treatment than male patients. Moreover, public health conditions generally focus more on the life cycle of women and breast cancer (33). During the COVID-19 pandemic, naturally, women tend to be more careful not to contract the coronavirus than men, so they choose to postpone visits even though they are sick.

The average cost per type of disease based on age has consistently decreased after the COVID-19 pandemic with the same pattern. Younger groups are generally healthier; and costs are concentrated among fewer individuals (34). This is one of the reasons why the financing of heart disease is getting bigger as the age group increases both in 2019 and 2020. At a young age, patients with cardiovascular disease have an ideal body fat composition and muscle mass and assume that their disease condition will be better. They have worsened when contracting the coronavirus in the hospital, thus choosing to postpone visits (35, 36). In contrast to older adults with cardiovascular disease who lose weight, most lose lean mass, which may contribute to an increased risk of cardiovascular events following weight loss, as body fat composition and muscle mass change with age, it is more urgent to visit health facilities consistently (37).

The phenomenon of a decrease in visits and a decrease in claims for National Health Insurance patients in cases of cardiovascular disease is not a reduction in the financing but is a pseudo-reduction, and there is a threat of an explosion in claims cases in future (38). Not to mention the condition of people's mental pressure from a wave of job loss cases, increasing poverty and unhealthy lifestyles threaten future cardiovascular disease cases (39, 40). A healthy lifestyle so as not to be exposed to expensive diseases is a substantial way to control the financing of cardiovascular disease.

A study in California in 2021 showed that during the COVID-19 pandemic, cardiovascular disease patients preferred telemedicine instead of attending health services which also aimed to control the cost of heart disease (41). This condition of using telemedicine is more common in developed countries (42). Meanwhile, in Indonesia, non-communicable disease control policies in this pandemic era have encountered more obstacles. More people delay visits to health facilities and wait for guidelines for visiting health facilities from expert doctors and specialists. The legal umbrella for telemedicine regulation has not yet been widely developed in Indonesia. Health facilities develop different telemedicine models by utilizing telecommunication application features, such as chatting or telephone to communicate with patients.

On the other hand, the Health Social Security Administration, as the operator of the National Health Insurance program in Indonesia, has developed a *JKN mobile* application that has the potential for telemedicine services to be guaranteed by the National Health Insurance (JKN). The advantages of the current Mobile JKN application are teleconsultation, the development of online prescribing, online queues, and health education for JKN participants (43). Technology-based screening, historical recording of participant claims history, and real-time data based on best practices are the directions for future development of the JKN program implemented by the Korean state since the COVID-19 pandemic (44). All of these efforts are carried out to prevent a spike in high-cost claim cases in future and prepare for another upcoming pandemic by strengthening the mobile JKN application. The Government of the Republic of Indonesia has also developed crowdfunding regulations for low-cost disease cases, so it is hoped that more savings will be made to prepare for financing claims for cardiovascular cases in future (45, 46). The next task of the Government and the community is to invite more people to adopt a healthy lifestyle to avoid cardiovascular disease and not aggravate existing disease conditions so that they do not become a burden on claims in future.

This study uses secondary data derived from the cost of health service claims from all hospitals in Indonesia. Secondary data are the given data; therefore, specific information cannot be obtained as complete as primary data.

The advantage of the study using national data is BPJS Health claims data so that it can describe financing conditions nationally. Meanwhile, the limitation of this study is the secondary data that are the given data; therefore, specific information cannot be obtained as complete as primary data. The available covariate adjustment factors are limited to the patient's age and sex. Further studies need to provide more adjustment factors to get a more accurate value of the difference in the claims, such as regional variations, service utilization time (months), and types of referral facilities.

## Conclusion

There has been a decrease in claims for cardiovascular care costs from before the COVID-19 pandemic, both in general and for all genders and age groups. Our findings show that the reduction in the cost of cardiovascular disease claims goes hand in hand with the decrease in the number of cardiovascular patient visits. Taking into account the factor of social restrictions during COVID-19, which affected the number of visits, when social restrictions are lifted or life returns to normal, the possibility of CVD visits will be the same as before COVID-19. Going forward, JKN needs to expand the scope of promotional and preventive services, especially for catastrophic diseases such as CVD, thereby reducing the burden on JKN in future. Health facilities that work with JKN need to prepare for better health service management in dealing with a pandemic, including digitizing services for patients, including CVD patients.

## Data availability statement

The data analyzed in this study is subject to the following licenses/restrictions: the raw data for this research cannot be accessed by the public. This is in accordance with data owner regulations (Social Security Administering Body for Health) that claims data is confidential and may not be consumed by the public. Officially we made a request on a clear proposal to the Data Management Division before the data delivered and used for our analysis and developing the manuscript. Requests to access these datasets should be directed to nugraheni_wp@yahoo.com.

## Ethics statement

This study had received ethical approval from the Health Research Ethics Committee, National Institute of Health Research and Development (HREC-NIHRD) with a letter number LB.02.01/2/KE.241/2021, on May 06, 2021. Written informed consent for participation was not required for this study in accordance with the national legislation and the institutional requirements.

## Author contributions

WPN, ER, and TR: conceptualization, methodology, writing—original draft, and writing—review and editing. RM: methodology, data curation, formal analysis, writing—original draft, and writing—review and editing. All authors have read and agreed to the published version of the manuscript.
